# Case Report: Should Regorafenib be prescribed as a continuous schedule in gastrointestinal stromal tumors? Three case reports on Regorafenib personalized schedule

**DOI:** 10.3389/fonc.2023.1190123

**Published:** 2023-05-31

**Authors:** Maria Susanna Grimaudo, Alice Laffi, Nicolò Gennaro, Roberta Fazio, Federico D’Orazio, Laura Samà, Licia Vanessa Siracusano, Federico Sicoli, Salvatore Lorenzo Renne, Armando Santoro, Alexia Francesca Bertuzzi

**Affiliations:** ^1^ Department of Oncology and Hematology, IRCCS Humanitas Research Hospital, Rozzano, Italy; ^2^ Department of Biomedical Sciences, Humanitas University, Pieve Emanuele, Italy; ^3^ Department of Radiology, Northwestern University, Chicago, IL, United States; ^4^ Department of Radiology, IRCCS Humanitas Research Hospital, Rozzano, Italy; ^5^ Department of Anatomical Pathology, IRCCS Humanitas Research Hospital, Rozzano, Italy; ^6^ IRCCS Humanitas Research Hospital, Department of Anatomical Pathology, Rozzano, Italy

**Keywords:** Regorafenib, sarcoma, GIST, case report, personalized therapy

## Abstract

**Introduction:**

Regorafenib is a tyrosine kinase inhibitor (TKI) approved in metastatic gastrointestinal stromal tumor (GIST), colorectal cancer, and hepatocarcinoma. Anyway, the toxicity profile of Regorafenib standard schedule is associated with poor compliance and a high rate of discontinuation. For this reason, there is a growing need for a Regorafenib personalized schedule emerging from the scientific community.

**Objective:**

The aim of this case series was to describe the experience of our sarcoma referral center with the continuous administration of Regorafenib as an alternative regimen to treat metastatic GIST patients.

**Methods:**

We retrospectively collected clinical, pathological, and radiological data of patients with metastatic GIST treated with daily personalized Regorafenib at a single tertiary referral center from May 2021 to December 2022.

**Results:**

We identified three patients fulfilling the inclusion criteria. The average follow-up since the start of Regorafenib was 19.1 months (12–25 months). All three patients had started a standard third-line Regorafenib schedule according to guidelines. The reasons for switching to a continuous schedule were as follows: exacerbation of symptoms during week-off treatment in the first patient, a serious adverse event (AE) in the second patient, and a combination of both conditions in the third. After switching, none of the patients reported severe AEs, and they improved control of tumor-related symptoms. Two of the patients experienced disease progression after 16 months (9 months of which is continuous schedule) and 12 months (8.1 months of which is continuous schedule) of Regorafenib, respectively; the third patient is still receiving continuous Regorafenib at the time of writing, with a progression-free survival of 25 months (14 months after the modified schedule start).

**Conclusion:**

With a similar efficacy and lower toxicities, a daily, personalized Regorafenib schedule seems to be a promising alternative to the standard regimen for metastatic GIST patients, including the frail ones. Further prospective analyses are needed to confirm the safety and efficacy of such regimen.

## Introduction

Despite overall rarity, gastrointestinal stromal tumors (GISTs) represent the most common subtype of mesenchymal tumors, with an incidence of 1.5/100,000 people/year worldwide. The median age at diagnosis is mid-60 years of age, with an equal distribution between men and women (1).

In the majority of cases, GISTs are diagnosed as a localized tumor, and only radical surgery is intended as a curative treatment. Perioperative treatment with a TKI (Imatinib mesylate, an inhibitor of KIT, PDGFRA, and ABL) is recommended in high-risk patients according to the risk assessment classifications and to the mutational pattern (2–6). In moderate-risk patients, perioperative treatment should be discussed with the patient (5, 6).

However, approximately 20% of patients present with metastases at diagnosis (7, 8) and up to 40% of patients who receive surgery tend to recur (9). In metastatic GIST, TKIs are the standard of care according to mutational status (6). Imatinib represents the first-line treatment for patients harboring an Imatinib-sensitive mutation (10), while Sunitinib constitutes the second-line treatment according to the results of the pivotal phase 3 trial (11).

Finally, Regorafenib represents the standard third-line treatment, based on the results of a phase 3 trial (GRID) (12), in which Regorafenib, at the dose of 160 mg daily orally for the first 3 weeks of each 4-week cycle (160 mg/day d1–21 q28), allowed a significant improvement of PFS versus placebo.

The efficacy of Regorafenib, an oral multi-TKI able to inhibit several kinases, including VEGFR1 to 3, TEK, KIT, RET, RAF1, BRAF, PDGFR and FGFR, is unfortunately afflicted by a high incidence of drug-related adverse events (AEs) and often requires personalized dose adaptations (13). Moreover, owing to the nature of GIST, some patients report an exacerbation of cancer-related symptoms during the week-off treatment (14). As an alternative schedule, Regorafenib continuous administration with a lower daily dose (120 mg/day continuously) but the same dose intensity in a 4-week cycle has been evaluated as feasible in a phase I study (15) and in a retrospective study (16).

Unfortunately, resistance to Regorafenib eventually occurs. Resistance to anti-angiogenetic agents can be a consequence of genetic/epigenetic modifications in cancer cells and/or in tumor endothelial cells (17). In order to avoid/delay resistance, several combinations of TKIs with other multiple angiogenetic drugs or immunotherapeutic agents are being studied.

Based on these biological and clinical data, we present our experience with a continuous schedule of Regorafenib.

## Methods

We retrospectively collected clinical, pathological, and radiological data of patients with metastatic GIST treated with continuous Regorafenib 120 mg/day after failure or intolerance to Imatinib and Sunitinib at Humanitas Research Hospital from May 2021 to December 2022. We anonymously collected data through the clinical records in an electronic database. All the patients signed an informed consent to the clinical research according to the institutional requirements.

## Results

**Figure 1:**
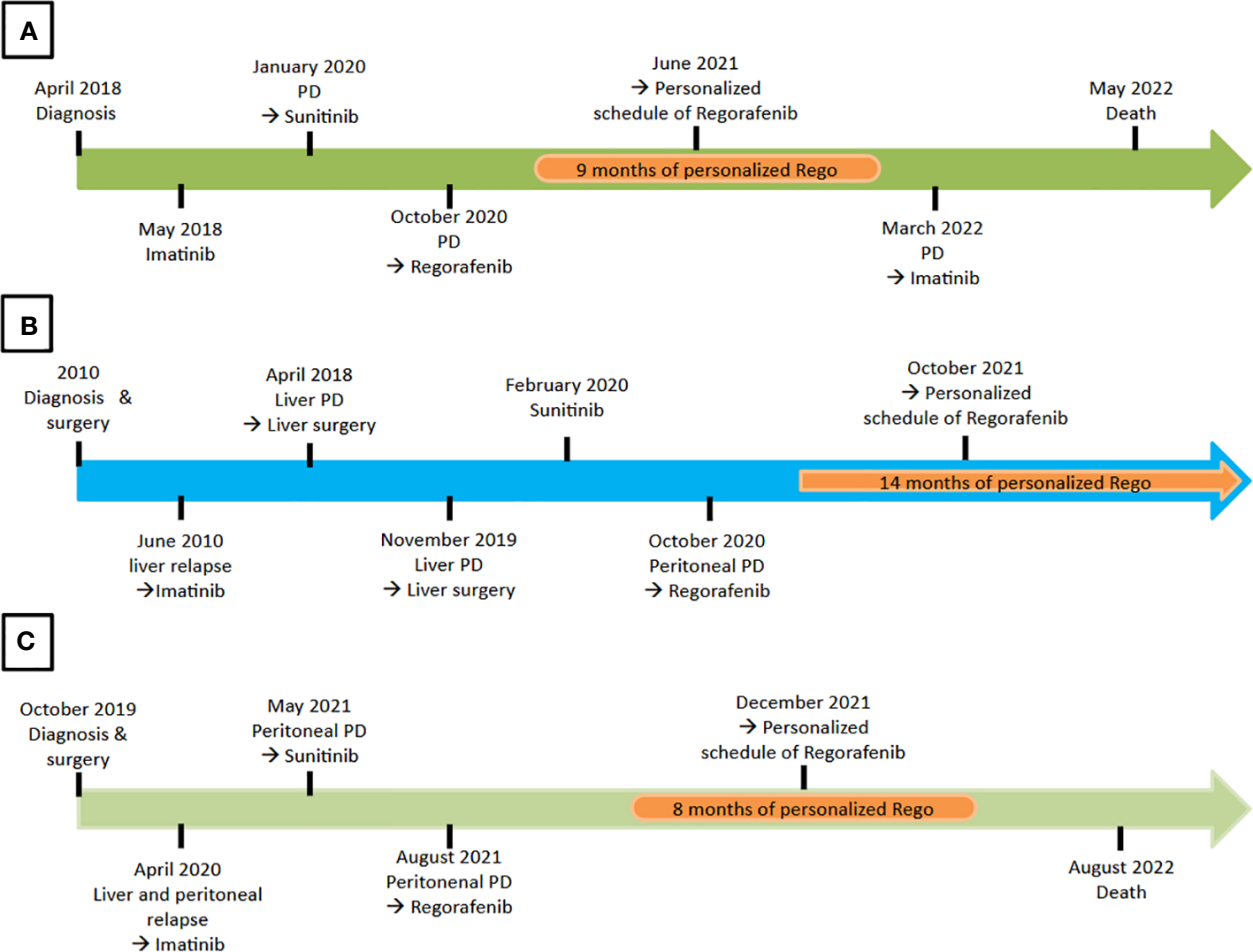
Timeline of treatments for each patient. **(A)** The patient 1 received 9 months of Regorafenib personalized treatment. **(B)** The patient 2 was still receiving personalized schedule of Regorafenib at the moment of the analysis. **(C)** The patient 3 received 8 months of Regorafenib personalized schedule.

We included three patients who fulfilled the inclusion criteria. In the same period of time, no other GIST patient received Regorafenib standard schedule in our institute. Patients’ characteristics are summarized in **Table 1**. The average follow-up since the start of Regorafenib was 19.1 months (12–25 months). All three patients had started a standard Regorafenib schedule. Below, we will briefly describe the clinical history of each patient. In **Figure 1**, we reported the timeline of each patient.

**Table 1:** Features of patients.

	Patient 1	Patient 2	Patient 3
**Age (years)**	50	53	62
**Sex**	M	M	M
**Site**	Small bowel	Stomach	Small bowel
**Mutational status**	KIT (exon 11)	KIT (exon 11)	Wild type
**Metastasis at diagnosis**	Yes	No	No
**Previous lines**	2	2	2
**PS ECOG at Regorafenib start**	3	1	3
**AEs ≥ G2—standard schedule**	No	Yes¹	Yes²
**AEs ≥ G2—modified schedule**	No	No	No
**Duration of modified schedule (months)**	9	14	8.1
**PFS with Regorafenib (months)**	17	25	12
**Subsequent lines**	Imatinib	No	No
**FU since start of Regorafenib (months)**	20.3	25	12
**FU since diagnosis (months)**	58	152	35
**Status**	Dead	Alive	Dead

M, male; PS, performance status; ECOG, Eastern Cooperative Oncology Group; Rego, Regorafenib; AEs, adverse events; PFS, progression-free survival; FU, follow-up.

¹ Hypothyroidism G2, anemia G3, nausea G2, anorexia G2, sialorrhea G2.

² Anemia G3, hypothyroidism G2.

### Patient 1

In April 2018 a 50-year-old man without relevant comorbidities accessed the Emergency Room due to abdominal increased volume, pain, and worsening of performance status (PS) according to the Eastern Cooperative Oncology Group (ECOG). An abdominal ultrasound showed a large mass of 17 × 14 cm and a CT scan confirmed the lesion associated with peritoneal localizations. A fine needle biopsy allowed the diagnosis of GIST presumably from the small bowel, with a mitotic index of 10/35 HPF (high power field). The tumor was stained positive for CD34, DOG1, and Caldesmon. The molecular pattern showed a mutation in KIT exon 11 [c.1657_1668del12; p.Y553_Q556del].

He received first-line therapy with Imatinib since May 2018, with an almost immediate symptoms relief and improvement of PS. After a month, a CT scan showed a trend to a reduction of the known lesions and the best overall response (BOR) as partial response (PR) occurred after 14 months of treatment, with subsequent disease progression (PD) after 21 months. Therefore, second line-therapy with Sunitinib was started. The BOR occurred after 4 months as stable disease (SD) according to Response Evaluation Criteria in Solid Tumors (RECIST) criteria version 1.1 (18), alongside a metabolic response. After 9 months, clinical and radiological PD occurred and PS of the patient was dramatically compromised (PS 3) because of complete bowel occlusion and intense pain. Despite the clinical situation and the need for total parenteral nutrition, we proposed a further treatment with Regorafenib, and the patient accepted.

Therefore, Regorafenib was gradually administered through the nasogastric tube with an initial reduced dose, obtaining a slow clinical improvement and a partial resolution of the bowel occlusion. After 1 month, the patient was receiving the standard dose of Regorafenib of 160 mg/day with the classic schedule; parenteral nutrition was progressively withdrawn in favor of oral nutrition.

The patient obtained as BOR a PR after 2 months of treatment, followed by SD. Nevertheless, the patient reported a significant worsening of abdominal symptoms during the week-off treatment, with an almost complete resolution at the restart of a new cycle of treatment. For this reason, according to few published data, the Regorafenib schedule was switched to a personalized regimen, administering 120 mg/day continuously. This schedule was well tolerated and provided an SD for a further 9 months. The patient reported no more symptoms, worsening periods, or AEs, indeed denoting subjective wellbeing. The overall PFS with Regorafenib was 17 months. At the time of PD, we proposed a rechallenge with Imatinib, but unfortunately, the patient’s clinical conditions dramatically worsened because of abdominal pain, bowel occlusion, and ascites, leading to death after less than 2 months from the initiation of Imatinib.

### Patient 2

A 53-year-old man without comorbidities except a low body mass index (BMI 17.15) came to our institute in 2018 with a diagnosis of gastric GIST with liver metastases. According to his medical history, he had undergone a total gastrectomy for a gastric GIST in another hospital in 2010. The histopathologic report had confirmed the diagnosis with a positivity for CD117, CD34, and DOG1 and a mitotic index of 18/25 HPF. Molecular analysis had shown a mutation in KIT exon 11 [W557-v559>Y]. The first CT scan performed after surgery had revealed a single liver metastasis, so a systemic treatment with Imatinib had been started, achieving a complete response (CR). After a treatment discontinuation of a few months, multifocal liver progression had occurred, so the patient had restarted Imatinib, obtaining disease control.

In April 2018, the patient, motivated by the long-lasting disease stability, accessed our institute to be evaluated for surgery and underwent multiple liver resections. The pathologic report detected four metastases of GIST, and the molecular pattern showed a mutation in KIT exon 11 [W557_V559>Y] and a new mutation in c-KIT exon 17 [D816G].

A new liver relapse occurred 2 months after surgery and Imatinib was gradually restarted, obtaining a metabolic CR after 1 month of treatment and a morphological SD after 6 months. After a further liver oligoprogression, the patient underwent a new liver resection, with histological confirmation of two metastases with the same mutational pattern of the previously resected ones. Nearly immediately after surgery, a liver relapse was detected and a new line of treatment with Sunitinib was started, with a PFS of 9 months and SD as BOR obtained after 2 months. At the time of PD, the CT scan detected pulmonary, liver, and new peritoneal lesions. Third-line treatment with Regorafenib was started 160 mg/day with the classic schedule. The patient interrupted the therapy after only 1 month because of a symptomatic (G2) hypothyroidism due to an autoimmune thyroiditis that warranted steroids and hormone replacement. Over the Regorafenib interruption, a stereotactic body radiotherapy was performed on all the known metastases. After 2 months, Regorafenib was cautiously restarted at 80 mg/day and then gradually increased until reaching a 160 mg/day standard schedule. Due to gastrointestinal toxicities (G2 nausea, G2 anorexia, and G2 sialorrhea), the dose was reduced to 120 mg/day d1–21 q28, with a morphologic SD and a metabolic CR after 3 months. As a consequence of an intestinal bleeding and G3 anemia that required blood transfusions, Regorafenib was interrupted. At the complete recovery, considering the medical history of the patient, the previous toxicities, and the low BMI, we proposed the resumption of Regorafenib with a personalized continuous schedule of 80 mg/day. This schedule has been well tolerated, without requiring new interruptions and with no more AEs other than G1 (hand skin reaction), obtaining SD. The patient is still progression-free after 25 months of treatment (14 months personalized schedule) and in subjective and objective good clinical conditions.

### Patient 3

A 63-year-old man came to our institute in 2021 for a second opinion for an ileal GIST with metachronous metastases. As significant comorbidities, he had had a myocardial infarction in 1977 and a stroke without neurological sequelae in 2007. In 2019, an abdominal large mass of 13 × 4.4 × 15 cm was detected due to abdominal pain, and in October 2019, surgery of the lesion was performed in another center. The pathology report diagnosed a high-risk [Mettienen et al. (2, 3)] ileal GIST with a positive staining for CD117, CD34, and DOG1 and a mitotic index greater than 5/25 HPF. Because of patient comorbidities, no adjuvant therapy was proposed and a liver and peritoneal relapse was detected after 6 months from surgery. First-line therapy with Imatinib 400 mg/day was proposed with initial SD, then PD after 9 months. Thus, Imatinib was increased to 800 mg/day, but a worsening of the clinical conditions occurred, leading the patient to the Emergency Room of our Institute with G3 acute heart failure that required hospitalization. A CT scan showed a further dimensional PD. In May 2021, after complete clinical recovery, a second-line treatment with Sunitinib was gradually started until the dose of 37.5 mg/day. The pathologic review of the histologic specimen confirmed the diagnosis, and the molecular analysis showed no mutations in the c-KIT gene. It was not possible to determine PDGRα gene status due to poor sample quality and quantity.

After 2 months of treatment, a severe gastrointestinal bleeding conditioning G3 anemia and acute kidney failure led to another hospitalization of the patient and discontinuation of the treatment. The CT scan showed an abdominal PD conditioning a severe bilateral hydronephrosis that required a right ureteral stenting and a left nephrostomy. Although the patient was suffering from abdominal pain and poor clinical conditions (PS ECOG 3), having achieved initial hematologic recovery and a clinical stabilization, in agreement with the patient in August 2021, standard schedule Regorafenib was started and progressively increased up to 120 mg/day d1–21 q28. The patient experienced a mild clinical improvement and radiologic SD, but reported a worsening of the symptoms (abdominal pain) over the week-off treatment. Moreover, owing to G3 anemia that required multiple blood transfusions, the treatment had to be interrupted and restarted at the recovery with a personalized schedule. Thus, we proposed a Regorafenib continuous schedule (120 mg/day), obtaining a good tolerance and no more AEs ≥G2, with the only AEs being G1 anemia and G1 hand–foot syndrome. Global clinical conditions significantly improved with subjective wellbeing, radiologic SD as BOR after 1 month, and metabolic PR after 5 months. A radiologic and clinical PD occurred after 12 months of therapy with Regorafenib (8 months personalized schedule) Regorafenib, and the patient was hospitalized for best supportive care. Unfortunately, his conditions, compromised by abdominal pain and bowel obstruction, did not allow any further treatment and he passed away 2 months after the discharge.

## Discussion

Regorafenib is approved as a third-line therapy in metastatic GIST, but it presents a challenging toxicity profile often requiring a personalization of therapeutic schedule. In the pivotal phase III GRID trial (12), 98% of patients receiving Regorafenib experienced at least one drug-related AE and 60% of the study population reported a G3 or higher AE [according to the Common Terminology Criteria for Adverse Events (CTCAE) v5.0]. Dose modifications were required in 72% of patients in the experimental arm, and 6% discontinued treatment due to AEs. This incidence of ≥G3 AEs was definitely more elevated than the one documented for Imatinib and Sunitinib, respectively, of 20.5% (10) and 20% (11).

Similar results were also reported in the phase III CORRECT trial (19) of Regorafenib in pretreated metastatic colorectal cancer, where the ≥G3 AEs occurred in 54% of patients, leading to a dose reduction or treatment discontinuation in 67% of cases.

The severe toxicity profile of Regorafenib across different cancers has also been highlighted by a systematic meta-analysis (13) that included seven studies and 2,099 patients: the authors registered 47% dose reductions, 57.2% dose interruptions, and 9.7% permanent discontinuations.

The published data were also confirmed in clinical practice as recently collected by Nannini et al. in a retrospective study conducted across several Italian sarcoma centers (20, 21), evaluating the real-life treatment strategies in 152 metastatic GIST patients on Regorafenib. Among them, only 32.2% received treatment at the standard dose, while the vast majority (67.8%) received a personalized dose/schedule, upfront or during the course of treatment. The most frequent dose modification was daily dose reduction to 120 mg or 80 mg maintaining the regular schedule, a scheme adopted in 54% and 21% of cases, respectively. Other dose adjustments affected only the schedule or both dose and schedule. The authors reported not only a complete or partial resolution of AEs in all patients receiving personalized treatment, but also a significant positive impact of this approach on PFS (mPFS 9.7 versus 5.6 months), observing also a trend towards OS improvement. Thus, the customization of a personalized regimen in the daily clinical practice allowed the achievement of a better disease control, probably due to the continuity of treatment.

A continuous schedule has been rarely adopted in Nannini et al. (20, 21), although it would be the best approach to maintain the dose intensity and to meet the unique GIST biology. Indeed, the kinase-addicted nature of this tumor requires a continuous suppression, as clinically described in a prospective study enrolling 57 GIST patients treated with Regorafenib (14), in which 26% of the patients treated with standard schedule suffered from an exacerbation of cancer-related symptoms during the week-off treatment, with a quick improvement at the new cycle start.

Imatinib and Sunitinib treatment schedule is based on this rationale. The discontinuation of Imatinib results in early disease progression (22), so that prosecution of treatment is continuously recommended. Despite the pivotal study of Sunitinib (11) with an intermittent schedule of 50 mg/day 4-weeks-on, 2-weeks-off, the equivalent dose-intensity regimen with 37.5 mg/day continuously was investigated and finally recognized as standard regimen in sarcoma referral centers (23). Indeed, even short suspensions of anti-VEGF agents lead to tumor regrowth, with full revascularization after 7 days of drug withdrawal (24).

Regarding Regorafenib, a continuous schedule was explored in a phase I study showing a favorable clinical activity and safety profile (15). In a subsequent retrospective study in GIST patients (16), 79% received a continuous dose of Regorafenib 120 mg/day. Overall, ≥G3 AEs were reported in 43% of patients, while treatment discontinuation due to AEs were registered remarkably in 21% of patients on classic schedule versus 14% of patients on continuous schedule.

Perhaps, to identify the correct dose and schedule of oral TKIs for every patient, a monitoring of drug plasma concentrations should be determined, as investigated in GIST patients on Imatinib (25) and recently re-proposed with Pazopanib (26). This personalized model might be appropriate but hardly feasible in clinical practice due to costs and complex techniques.

Resistance to anti-angiogenic therapies is a significant challenge in oncology, as it leads to a lack of response and disease progression. It can develop due to genetic/epigenetic changes in cancer cells or endothelial cells and it can imply different mechanisms of tumor angiogenesis, as intussusceptive microvascular growth, vasculogenic mimicry, and vascular co-option. To overcome resistance, alternative therapeutic strategies have been explored, such as combining multiple anti-angiogenic drugs or anti-angiogenic drugs with immune checkpoint inhibitors, as it has been successfully done in other angiogenesis-dependent tumors such as renal cancer (17). In GIST, this combination is being studied in different clinical trials (27). Potentially, in the future, pan-omics profiling could allow physicians to identify the most suitable treatment for each patient (17).

As for personalization of the cure, our cases support the previously reported data on continuous schedule Regorafenib, showing a comparable efficacy through a steady suppression of the oncogenic pathways and guaranteeing a better tolerance even in frail patients who had experienced serious AEs with standard schedule Regorafenib.

Certainly, our analysis presents several limitations. Being a retrospective analysis, data can be affected by bias or missing. Secondly, the study was conducted at a single tertiary referral center, which may limit the generalizability of the findings to other populations or healthcare settings. Thirdly, the sample size is small, preventing us from applying statistical analysis and drawing robust conclusions. Lastly, the short follow-up may limit the assessability of long-term safety and efficacy of continuous schedule Regorafenib in this setting.

Also, a personalized approach itself presents some limitations, such as the physician team’s expertise in identifying patients that could benefit the most from the schedule adjustment and the difficult generalizability of personalized schedules.

On the other hand, a personalized schedule allows to take into account the patient’s needs and perspective, leading to an increased awareness of his cure plan and a better compliance.

Our study provides initial evidence for the potential benefits of a continuous, personalized Regorafenib schedule, and these findings suggest that such a regimen may be a promising alternative to the standard, with similar efficacy and lower toxicities. However, further prospective studies with larger sample sizes and longer follow-up periods are needed to confirm the safety and efficacy of this treatment approach.

## Data availability statement

The raw data supporting the conclusions of this article will be made available by the authors, without undue reservation.

## Ethics statement

The studies involving human participants were reviewed and approved by Internal Humanitas Research Hospital IRCCS committee. The ethics committee waived the requirement of written informed consent for participation.

## Author contributions

Conceptualization and first draft: AFB, MSG, RF. Data collection: MSG, RF, AL. Review: AFB, MSG, NG, AL, RF, LS, LVS, FD, FS, SLR, AS. Supervision: AFB, AS, AL. All authors contributed to the article and approved the submitted version.
